# Differences between COVID-19-induced acute kidney injury and chronic kidney disease patients

**DOI:** 10.1590/2175-8239-JBN-2021-0161

**Published:** 2022-02-28

**Authors:** Gustavo Aroca-Martínez, Carlos G. Musso, Lil Avendaño-Echavez, María Vélez-Verbel, Stefani Chartouni-Narvaez, Sandra Hernandez, Mauricio Andres Hinojosa-Vidal, Zilac Espitaleta, Andrés Cadena-Bonfanti

**Affiliations:** 1 Universidad Simón Bolívar, Faculdade de Ciências da Saúde, Barranquilla, Colômbia.; 2 Clínica de la Costa, Departamento de Nefrologia, Barranquilla, Colômbia.; 3 Instituto Universitario del Hospital Italiano de Buenos Aires, Departamento de Fisiologia, Buenos Aires, Argentina.; 4 Hospital Italiano de Buenos Aires, Departamento de Investigação, Buenos Aires, Argentina.; 5 Universidad Libre, Faculdade de Ciências da Saúde, Barranquilla, Colômbia.

**Keywords:** COVID-19, Acute Kidney Injury, Renal Insufficiency, Chronic, COVID-19, Injúria Renal Aguda, Insuficiência Renal Crônica

## Abstract

**Introduction:**

This article describes the main differences between COVID-19-induced acute kidney injury (AKI-COVID19) in patients with previous normal renal function (AKI-NRF) and those with chronic kidney disease (AKI-CKD) treated in a high complexity clinic in Barranquilla (Colombia).

**Material and Methods:**

The patients included in this study (n: 572) were those with a positive diagnosis of COVID-19 confirmed by detection of a positive PCR for SARS-CoV-2. Of these patients, 188 developed AKI during their hospital stay. Patients’ epidemiological data, serum parameters, and clinical frailty status were recorded. Statistical analysis and comparison among AKI-NRF, AKI-CKD, and non-AKI patients were performed.

**Results:**

The incidence of COVID-19-induced AKI was 33%, with the majority classified as AKIN 1, 16% requiring renal replacement therapy, and AKI-COVID19 mortality of 68%. A significantly higher prevalence of hypertension, cardiac disease, and serum reactive C-protein and lower albumin values in AKI-CKD patients was recorded. Mortality rate, invasive ventilation requirement, and D-dimer levels were significantly higher in AKI-NRF patients:

**Conclusion:**

Different clinical patterns between AKI-NRF and AKI-CKD were documented.

## Resumo

**Introdução:**

Este artigo descreve as principais diferenças entre a lesão renal aguda induzida por COVID-19 (LRA-COVID19) em pacientes com função renal normal prévia (LRA-FRN) e aqueles com doença renal crônica (LRA-DRC) atendidos em uma clínica de alta complexidade em Barranquilla (Colômbia).

**Material e Métodos:**

Os pacientes incluídos neste estudo (n: 572) foram aqueles com um diagnóstico positivo de COVID-19 confirmado pela detecção de PCR positivo para SARS-CoV-2. Destes pacientes, 188 desenvolveram LRA durante sua internação. Foram registrados os dados epidemiológicos, os parâmetros séricos e o estado de fragilidade clínica dos pacientes. Foram feitas a análise estatística e a comparação entre pacientes com LRA-FRN, LRA-DRC, e pacientes sem LRA.

**Resultados:**

A incidência de LRA induzida por COVID-19 foi de 33%, com a maioria classificada como AKIN 1, 16% exigindo terapia renal substitutiva, e a mortalidade por LRA-COVID19 foi de 68%. Foi registrada uma prevalência significativamente mais alta de hipertensão, doença cardíaca e proteína C reativa sérica e valores mais baixos de albumina em pacientes com LRA-DRC. A taxa de mortalidade, a necessidade de ventilação invasiva e os níveis de dímero-D foram significativamente mais altos em pacientes com LRA-FRN.

**Conclusão:**

Foram documentados padrões clínicos diferentes entre LRA-FRN e LRA-DRC.

## Introduction

In late 2019, a new type of acute respiratory insufficiency was described in Wuhan, China. It was later found to be caused by a new variety of coronavirus (SARS-CoV-2), and the disease which it caused was named COVID- 19^1,2^. Even though COVID-19 affects mostly the patient’s respiratory system, this condition can also alter other organs such as kidneys, intestines, bone marrow, heart, and nervous system^3^.

Regarding renal damage, an incidence of 3-9% was reported, ranging from urinary disorders such as albuminuria (34%), proteinuria (63%), hematuria (27%), proteinuria with hematuria (44%), to an increase in serum creatinine and urea levels due to acute kidney injury (AKI) (14-27%)^4^.

Several mechanisms have been implicated in COVID-19-induced kidney damage. Some are due to the direct deleterious effect of the virus on podocytes, tubular and endothelial cells, while others are involved in indirect kidney damage induced by reduced perfusion (shock), hypoxia (respiratory insufficiency), microangiopathy (disseminated intravascular coagulation: DIC), pigmenturia (rhabdomyolisis), immunological renal damage (cytokine storm), and drug toxicity (AINEs)^5-11^.

Likewise, AKI-COVID19 has been reported to occur mainly in critically ill patients, in whom it is an additional factor for poor prognosis, increasing mortality by up to 92%^6-8^. Among the risk factors favoring its appearance are the presence of oncologic disease, sepsis, heart failure, and DIC^8^. However, as far as we know, the literature has not yet described whether there are differences between AKI-COVID19 in patients without previous kidney disease and those with CKD. Thus, it was decided to perform a prospective study with the objective of exploring whether there was a significant difference in inflammatory response and mortality between *de novo* AKI COVID-19 patients and AKI COVID-19 patients, who previously suffered from CKD, who were assisted during the first pandemic wave (2020) in the Clínica de la Costa, Barranquilla (Colombia).

## Material and Methods

This was a prospective observational study with patients treated at the emergency room in the Clínica de la Costa, Barranquilla (Colombia) from April 01 to July 11, 2020 due to suspicion of COVID-19. Patients with confirmed COVID-19 diagnosis by positive polymerase chain reaction (PCR) test were included in the study. Additionally, data from admitted COVID-19 patients who developed AKI during their admission was collected in an electronic record specifically designed for this purpose.

The AKI patient group was divided into two subgroups: those who had previous normal renal function (AKI-NRF) and those who had previous chronic kidney disease (AKI-CKD). CKD diagnosis was obtained from the patient’s electronic clinical record, confirmed by at least one of the following chronic kidney alterations: reduced glomerular filtration rate (≤ 90 mL/min/1.73 m^2^) with abnormal urinalyses (dysmorphic hematuria, and/or proteinuria), and/or abnormal renal ultrasound (multiple cysts, kidney size reduction, parenchymal hyperechogenicity, renal cortex-medulla border loss)^12^.

Finally, clinical and biochemical parameters were compared among AKI-NRF, AKI-CKD, and NON-AKI patients, represented by those individuals who did not suffer from AKI.

Sample handling and processing for SARS-CoV-2 diagnosis were conducted in accordance with the reverse transcription real-time PCR (RT-PCR) guidelines (Diagnostic detection of 2019-nCoV by real-time RT-PCR Charité Virology, Berlin, Germany)^10^.

People with previous normal kidney function were defined as those with normal glomerular filtration rate (> 90 mL/min/1.73 m^2^), normal urinalysis, normal renal ultrasound and no personal history of renal disease at admission.

AKI in people with previous normal function was defined as an increase in creatininemia of >0.3 mg/dL compared with their basal serum creatinine value at admission. In addition, each documented episode of AKI was classified according to the AKIN criteria (Table 1)^11^. AKI in individuals previously diagnosed with CKD was defined as an increase in creatininemia of ≥ 1.5 times respect compared with their basal serum creatinine value at admission^11^.

**Table 1:** Acute kidney injury (AKI) stages (KDIGO 2012)

AKIN stage	Definition
**1**	Increase in serum creatinine levels >0.3 mg/dL or 1.5-1.9 times the baseline creatinine value, and/or decreased urine output to 0.5 mL/kg/h for 6 h.
**2**	Increase in serum creatinine levels 2-2.9 times the baseline creatinine value, and/or decreased urine output to 0.5 mL/kg/h for 12 h.
**3**	Increase in serum creatinine levels 3 times the baseline creatinine value, increase of serum creatinine >4 mg/dL, initiation of RRT and/or decreased urine output to 0.3 mL/kg/h for 24 h or anuria for 12 h.

*sCr: Serum creatinine; RRT: Renal replacement therapy; KDIGO: Kidney Disease Improving Global Outcomes.*

NON-AKI individuals were defined as admitted patients with no AKI, AKI-NRF, or AKI-CKD during their admission.

The following parameters were obtained daily from each patient during his/her hospitalization: serum creatinine, urea, electrolytes, blood count, bilirubin, transaminases, lactate dehydrogenase (LDH), troponin, C-reaction protein (CRP), ferritin, D-dimer, and coagulation parameters: partial thromboplastin time (PTT), prothrombin time (PT), and international normalized ratio (INR). In addition, presence of proteinuria, hematuria, and/or leukocyturia, as well as the degree of pre-existing frailty status by the Clinical Frailty Scale (CFS) were also documented (Table 2)^12^.

**Table 2:** Clinical frailty scale

1-Very fit	Robust, active, energetic, and motivated persons. These persons commonly exercise regularly. They are among the most fit for their age.
2-Well	Persons who have no active diseases and no symptoms, but who are less fit than those in the previous category. They often exercise or are very active from time to time.
3-Managing well	Persons whose medical problems are well controlled but are not regularly active beyond routine walking.
4-Vulnerable	While not dependent on others for daily help, their symptoms often limit activities. A common complaint is being “slowed up” and/or being tired during the day.
5-Mildly frail	These persons often have more evident slowing and need help with more complex activities (managing their finances, medicines, transportation, and heavy housework).
6-Moderately frail	These persons need help with all outdoor activities. Indoors, they need help with cleaning and often have problems climbing stairs. They also need help bathing and may need minimal assistance to get dressed.
7-Severely frail	Completely dependent for personal care either due to physical or cognitive reasons. Even so, they seem stable and not at high risk of dying.
8-Very severely frail	Completely dependent, and close to the end of their life (within 6 months).
9-Terminally ill	Approaching the end of life. This category applies to anyone with a life expectancy <6 months, who is not evidently fragile.

The quantitative variables were summarized as mean and standard deviation (SD) or median and interquartile range. Qualitative variables were summarized as absolute and percent relative frequencies. Comparisons of quantitative variables between groups defined by the presence of kidney injury were carried out using the ANOVA test for comparison of means in independent samples or the Mood test for comparison of medians. Comparison of qualitative variables was carried out using the Chi-Square association test or Fisher’s exact test. The magnitude of associations and correlations were determined using Pearson’s linear correlation coefficient, Kendall’s tau coefficient, or odds ratios, depending on the type of variables analyzed. Associated probability values less than 0.05 were considered statistically significant.

The statistical software used for this analysis was R Foundation for Statistical Computing, version 4.0.1; Vienna, Austria.

This study was approved by the Ethical Committee of Clinica de la Costa, Barranquilla (Colombia), and informed consent was obtained from all patients.

## Results

Of the 720 individuals evaluated at the emergency room of the Clínica de la Costa, Barranquilla (Colombia) from April 01 to July 11, 2020, for suspicion of COVID-19, 572 were admitted with confirmed diagnosis of SARS-CoV-2 infection (positive PCR test). Most were male (59%) with a median age of 55 years (range: 37-70). Hypertension was the most frequent comorbidity (36%), followed by obesity (23%), diabetes mellitus (18%), heart disease (5%), and chronic obstructive pulmonary disease (COPD) (9%). With respect to their clinical status, almost all were robust individuals (97%) who had an optimal functional score (CFS 1: 76%). Meanwhile, a minority of patients was frail (3%), with mild frailty score (CFS: 4-5).

The most frequent symptom presented by the patients at the beginning of hospitalization was elevated body temperature (65%), followed by dyspnea (58%), and non-productive cough (53%), while hypogeusia, anosmia, and productive cough were their least frequent symptoms (10%).

Forty percent of admitted COVID-19 patients required assisted mechanical ventilation (MV) (20% invasive MV and 20% non-invasive MV) and 9% required renal replacement therapy. Finally, the presence of AKI (p: 0.04), patient age (p: 0.03), and frailty status (p: 0.02) showed significant direct correlation with patient mortality.

During patients’ hospital stay (median: 4 days), 188 COVID-19 patients developed AKI (incidence: 33%), with 149 (26%) having a previous normal renal function (AKI-NRF), while 39 (7%) having a previously CKD diagnose (AKI-CKD). From the 43 admitted CKD patients, most (91%) developed AKI. Besides, there was a predominance of men, elderly (≥ 60 years), frail (CFS ≥ 4), diabetic, obese, COPD individuals in the AKI group (AKI-NRF and AKI-CKD subgroups) compared with the NON-AKI group (n: 380) (Table 3).

**Table 3:** Mean age, gender, and chronic conditions of the groups

n: 570	AKI-NRF (26%)	AKI-CKD (8%)	NON-AKI (66%)	P value
Age (mean, range years)	64 (53-74)	63 (55-77)	48 (33-65)	1: <0.0001 2: NS 3: <0.0001
Male	74%	70%	51%	1: <0.0001 2: NS 3: <0.0001
Frailty	9%	7%	0,3%	1: <0.0001 2: NS 3: <0.0001
Hypertension	54%	79%	25%	1: <0.0001 2: 0,02 3: <0.0001
Obesity	31%	30%	18%	1: 0.015 2: NS 3: 0.015
DM	26%	25%	14%	1: 0.002 2: NS 3: 0.002
COPD	16%	16%	6%	1: 0.0015 2: NS 3: 0.0015
CD	8%	18%	3%	1: <0.0001 2: <0,0001 3: <0.0001

AKI-NRF: acute kidney injury in previously normal renal function individuals; AKI-CKD: acute kidney injury in chronic kidney disease patients; COPD: chronic obstructive pulmonary disease; DM: diabetes mellitus; CD: cardiac disease; NS: non-significant Bonferroni test comparisons: AKI-NRF vs NON-AKI (1), AKI-NRF vs AKI-CKD (2), AKI-CKD vs NON-AKI (3).

The prevalence of hypertension and cardiac disease was significantly higher in the AKI-CKD group compared with the AKI-NRF group, and even higher compared to the NON-AKI group (Table 3). However, there was a higher mortality rate in the AKI-NRF group (69%) compared with the AKI-CKD group (56%), but it did not reach statistical significance (p: 0.09). The mortality rate in the AKI group compared with the NON-AKI group (16%) was significantly higher (p: <0.0001).

It is worth mentioning that in AKI-NRF individuals the most frequent AKI stage was AKIN 1 (53%), followed by AKIN 3 (29%) and AKIN 2 (18%). However, the highest AKI degree reached during hospitalization was AKIN 3 (42%), followed by AKIN 1 (38%). Regarding the transition among AKI levels, of patients who were initially AKIN 1, 13% of them later reached AKIN 2 level and 15% reached AKIN 3 level, while among patients who were initially AKIN 2, 23% later reached AKIN 3. Finally, 48% of patients who suffered from AKI during hospitalization presented their highest AKIN degree in the intensive care unit.

Among the main symptoms presented by COVID-19 patients at admission, the most significantly associated with this condition were dyspnea (p: <0.0001), fatigue (p: 0.0090), and headache (p: 0.0054). The presence of dyspnea and fatigue was more frequently observed in AKI-COVID19 patients, either AKI-NRF (82%) or AKI-CKD (77%) compared with NON-AKI patients (47%), while headache was more frequently documented in NON-AKI patients (25%) compared with AKI patients (13%). The hospitalization period was significantly longer for AKI patients (8 days, range 3-14) than in NON-AKI patients (2 days, range: 1-6) (p: <0.0001).

Regarding artificial organ support requirement, invasive ventilation support was slightly more required in the AKI-NRF group compared with the AKI-CKD group (p: 0.05), but much more required compared with the NON-AKI group (p: <0.001). Conversely, non-invasive ventilation support was significantly more required in the NON-AKI group compared with the AKI-CKD and AKI-NRF groups (p: <0.001) (Table 4).

**Table 4:** Requirement of vital organ artificial support in the groups

n: 570	AKI-NRF (26%)	AKI-CKD (8%)	NON-AKI (66%)	P value
Non-Invasive Ventilation	32%	39%	73%	1: <0.0001 2: NS 3: <0.0001
Invasive Ventilation	68%	60%	27%	1: <0.0001 2: 0,05 3: <0.0001
Dialysis	22%	42%	0%	2: <0.0001

AKI-NRF: acute kidney injury in previously normal renal function individuals; AKI-CKD: acute kidney injury in chronic kidney disease patients; NS: non-significant Bonferroni test comparisons: AKI-NRF vs NON-AKI (1), AKI-NRF vs AKI-CKD (2), AKI-CKD vs NON-AKI (3)

With regards to hemodialysis, intermittent modality (67%) was the most commonly used modality, being more significantly required by AKI-CKD patients (42%) than by AKI-NRF (22%), p: <0.0001 (Table 4).

There was no significant difference among the studied groups in laboratory parameters except for serum creatinine and urea levels at AKI diagnosis time, which showed the highest level in the AKI-CKD group, followed by the AKI-NRF group, and the lowest values were for the NON-AKI group (p: <0.001) (Table 5).

**Table 5:** Main serum parameters in the groups

n: 570 median, range	AKI-NRF (26%)	AKI-CKD (8%)	NON-AKI (66%)	P value
SCr (mg/dL)	1.5 (1.1-2.3)	4.3 (1.8-7.3)	0.7 (0.6-0.9)	1: <0.0001 2: <0.0001 3: <0.0001
SU (mg/dL)	92 (51-145)	105 (58-156)	30 (21-41)	1: <0.0001 2: NS 3: <0.0001
SG (mg/dL)	136 (101-196)	123 (79-181)	102 (84-139)	1: 0.04 2: NS 3: NS
SA (g/dL)	2.4 (2.2-3.4)	2.9 (1.8-3.0)	3.3 (2.9-4.2)	1: 0.03 2: 0.05 3: NS
Leuco (mm^3^)	12.450 (8.575-18.975)	10.200 (6.450-14.925)	9.100 (6.400-12.950)	1: 0.0003 2: 0.03 3: NS
LDH (UI/L)	562 (366-917)	492 (294-824)	312 (248-461)	1: <0.0001 2: 0.06 3: NS
CRP (mg/dL)	25 (6.-35)	8 (2-9)	5.9 (1-11)	1: 0.002 2: 0.06 3: NS
Ferritin (Ug/L)	1070 (858-2014)	892 (327-1875)	530 (253-946)	1: 0.03 2: 0.06 3: NS
D-Dimer (ng/mL)	4487 (1032-6469)	4050 (990-5903)	1015 (329-4798)	1: 0.03 2: 0.05 3: 0.03

AKI-NRF: acute kidney injury in previously normal renal function individuals; AKI-CKD: acute kidney injury in chronic kidney disease patients; SCr: serum creatinine; SU: serum urea; SG: serum glucose; SA: serum albumin; Leuco: leucocytes; CRP: C-reactive protein; NS: no significant Bonferroni test comparisons: AKI-NRF vs NON-AKI (1), AKI-NRF vs AKI-CKD (2), AKI-CKD vs NON-AKI (3)

Serum glucose and most inflammatory parameters (leucocytes, LDH, and ferritin) were significantly higher in AKI patients compared with NON-AKI patients. Serum CRP levels were slightly higher and albumin was lower in the AKI-NRF group than in the AKI-CKD group. Serum D-dimer was slightly higher in the AKI-NRF group than in the AKI-CKD group. The NON-AKI group showed significantly lower serum CRP and D-dimer, as well as higher serum albumin value compared with the AKI group (Table 5).

In addition, AKI-COVID19 showed a significant association with patient negative outcome (p: <0.0001), since in-hospital deaths were more frequent in AKI patients (AKI-NRF and AKI-CKD), than in NON-AKI patients (68.46%, 55.81% and 15.49%, respectively). In this sense, death risk was 11.83 times higher in AKI patients compared with NON-AKI patients (OR: 11.86; 95% CI: (7.58; 18.44)) and 6.74 times higher among CKD patients compared with NON-AKI (OR: 6.74; 95% CI: (3.47; 13.06)).

## Discussion

In our study, AKI-COVID19 incidence was 33%, being 26% in previously normal kidney function individuals and 7% in CKD patients. As expected, most CKD patients who were affected by COVID-19 (91%) developed AKI, probably due to their increased susceptibility to kidney damage. AKI-COVID19 incidence in other reports ranges between 5 and 76%. This notorious difference could be due to diverse epidemiological characteristics (age, comorbidities, etc.) and disease severity (ambulatory, critical care, etc.) among the populations studied^13-16^.

As reported in previous literature, AKI-COVID19 appeared within the first week of hospitalization in our study.^15^ Likewise, its predominance in males and in older, frail, diabetic, obese, COPD individuals was also documented in other reports.^14.^ It has been suggested that these chronic inflammatory conditions contribute to the deleterious effect of the immune system overreaction (cytokine storm), which characterizes COVID-19 disease^9,16-22^.

Increased serum interleukin-6, ferritin, transferrin, D-dimer, fibrinogen, and CRP are among the most documented characteristic of systemic inflammatory syndrome associated with SARS-CoV-2 acute infection. Ferritin is an intracellular iron storage protein that appears in blood during systemic stress and stimulates the innate immunity cells that trigger the cytokine storm.^23-37^

It is worth mentioning that hypertension and cardiac disease were significantly more common in the AKI-CKD group than in the AKI-NRF group. This phenomenon could be explained by the increased prevalence of hypertension and cardiopathy in CKD patients. In this sense, both conditions can induce kidney alteration (e.g., nephroangiosclerosis and cadiorenal syndrome, respectively) or be the consequence of excessive sodium retention and volume overload due to chronic nephropathy.^23-39^

Furthermore, there was a trend toward higher mortality, higher need for invasive mechanical ventilation, higher serum inflammatory markers (CRP, ferritin, LDH, leukocytes) and serum D-dimer levels (a marker of thrombin generation and fibrinolysis related to endothelial damage) in the AKI-NRF group compared to the AKI-CKD group. At the same time, AKI-CKD patients had significantly higher renal replacement requirement than AKI-NRF (42 vs. 22%, respectively). This phenomenon could be explained by the following hypothesis: both AKI subgroups had similar advanced age, frailty and comorbidities, which explain their worse evolution compared with the younger, robust, and less comorbid individuals in the NON-AKI group. However, the relatively better evolution of the AKI-CKD patients compared with the AKI-NRF patients could be explained by their chronic immunosuppression status associated with chronic nephropathy, which could avoid hyperinflammatory reaction (cytokine storm); the cornerstone of COVID-19-induced damage.^38,39^

Concerning the documented AKI subtype (AKIN) at the moment of renal injury diagnosis, the most prevalent class was AKIN 1 and the least frequent was AKIN 2, which is in line with some previous reports. However, other studies reported that AKIN 3 was the most common class, followed by AKIN 1.^14,15^ This discrepancy could be attributed to different times of AKI stage at which the condition was diagnosed in each study, i.e., the earlier the diagnosis was made, the lower the reported AKIN score.

Renal replacement therapy (RRT) was required in 22% of the AKI-NRF patients and in 42% of AKI-CKD patients. This is in line with what has been reported in literature, where RRT use oscillates between 14-38%.^14,15^

Many mechanisms have been proposed for why COVID-19 might predispose to AKI appearance. They can be summarized as follows:^9,16-20^

- Pre-renal: real hypovolemia (volume depletion), effective hypovolemia (heart failure, sepsis), microangiopathy (coagulopathy).
- Renal: viral cytotoxic effect, cytokine damage (hyperinflammation), hypoxia (pulmonary distress), drug toxicity, collapsing glomerulopathy.
- Intra-renal obstruction: myoglobin (rhabdomyolysis).

With regards to symptoms at admission, dyspnea and fatigue were more frequent among AKI patients, for both AKI-NRF (82%) and AKI-CKD (77%) compared with NON-AKI patients (47%). This could be explained by the appearance of worst lung COVID-induced compromise and/or lung congestion in AKI patients. The latter condition could be explained by inadequate volume retention secondary to reduced glomerular filtration rate.

Serum glucose and inflammatory (high serum leukocytes and ferritin levels) or cell destruction (high serum LDH level) parameters were significantly higher in AKI patients compared with NON-AKI patients (Table 5). These findings are reasonable because, first diabetes mellitus was more prevalent in AKI patients and, second, the inflammatory status, represented by the presence of higher leucocyte, ferritin, and LDH levels, was probably worse in patients who developed AKI than in NON-AKI patients.

As expected, the NON-AKI group, which was the least sick, had the highest serum albumin levels and the lowest levels of serum inflammatory parameters, invasive MV requirement, and mortality.

COVID-19-associated mortality in AKI-NRF, AKI-CKD, and NON-AKI patients was 68.46, 55.81, and 15.49%, respectively. COVID-19 mortality in previous reports ranged from 16.1 to 62%.^13-15,24^

In our study, the variables that showed a significant direct correlation with mortality secondary to COVID-19 were: age, frailty, and presence of AKI. Moreover, length of hospitalization was significantly longer in AKI patients than in NON-AKI patients (p: <0.0001). This findings could be explained by the fact that these patients require more time to cope with SARS-CoV-2 infection because of several reasons, such as insufficient immune response (ageing, frailty) and excessive inflammation (AKI).

Finally, the comparisons between AKI-NRF and AKI-CKD with a trend toward significance (borderline p-value) could be due to the relatively small number of CKD patients in this study (n: 43). Future studies could clarify the real significance of these findings.

## Conclusion

This study reported a trend toward a higher inflammatory response and significantly higher mortality rate in *de novo* AKI in COVID-19 patients than in AKI COVID-19 patients who previously suffered from CKD.
